# Immediate Effects of Single-Session High-Velocity Training for Lateral Trunk Movement on Gait Function in Early Postoperative Patients after Total Hip Arthroplasty: A Nonrandomized Controlled Trial

**DOI:** 10.3390/healthcare10020256

**Published:** 2022-01-28

**Authors:** Keisuke Honma, Yuki Yano, Saki Yamamoto, Toshimitsu Ohmine, Hideyuki Wanaka, Kazuma Senzaki, Atsuki Kanayama, Hiroyuki Oonishi, Akira Iwata

**Affiliations:** 1 Graduate School of Comprehensive Rehabilitation, Osaka Prefecture University, Habikino 583-8555, Japan; tenniyuki@gmail.com (Y.Y.); yamamotos@rehab.osakafu-u.ac.jp (S.Y.); toshimitsu0605@gmail.com (T.O.); kazuma66gam@gmail.com (K.S.); kanayama@rehab.osakafu-u.ac.jp (A.K.); 2 Department of Rehabilitation, Tominaga Hospital, Osaka 556-0017, Japan; minichan07kh@gmail.com; 3 Department of Rehabilitation, Kobe Rosai Hospital, Kobe 651-0053, Japan; h.wanaka0531@gmail.com

**Keywords:** keyword gait speed, exercise, hip

## Abstract

Background: Total Hip Arthroplasty (THA) is an effective method for relieving pain and improving gait function. However, THA patients demonstrate slow gait speed at discharge. Rehabilitation programs after THA require the immediate improvement of gait speed early in the postoperative period. To examine the immediate effects of seated side tapping training (SSTT), which focuses on lateral trunk movement and movement velocity, on gait function in early postoperative THA patients, the methods were as follows: The SSTT group performed five repetitions of a task in which they moved their trunks laterally to alternately touch markers to their left and right side as quickly as possible 10 times in a seated position. One set of SSTT lasted approximately 3 min. The control group rested in a seated position for 10 min. Results: Significant interactions were observed for gait speed, stride time, and stride time coefficient of variability. The SSTT group demonstrated significant pre-post-intervention improvement in gait speed, stride time, and coefficient of variability. Conclusions: SSTT improved both gait speed and gait stability and can be performed easily and safely. Therefore, single-session high-velocity trunk training may be an effective method to improve gait function immediately in early postoperative THA patients.

## 1. Introduction

Total hip arthroplasty (THA) is an effective method for relieving pain and improving function, and activities of daily living [1,2]. The duration of hospitalization after THA has been shortened by the development of minimally invasive procedures, reductions in medical fees, and intensive physical therapy starting shortly after surgery [3,4]. However, THA patients demonstrate a very slow gait speed of 0.19–0.43 m/s at discharge [5] and improving gait function after THA by traditional rehabilitation programs requires a period of 6 to 12 weeks [6,7,8,9,10]. Since a gait speed of 1.0 m/s or more is necessary to live safely [11], rehabilitation programs after THA require the immediate improvement of gait speed early in the postoperative period.

Gait speed is more closely related to muscle power (strength × velocity) than muscle strength [12]. Some previous studies have indicated that high-velocity training increased muscle power in the very short term [13,14,15]. Therefore, high-velocity training of hip joint muscles might have immediate effects on gait speed in patients with THA. However, high-velocity training of the hip joint is difficult for early postoperative patients with THA due to pain or fear of movement. Seated side tapping training (SSTT), which focuses on high-velocity movement of the trunk, has been shown to improve gait speed in the early postoperative period of total knee arthroplasty (TKA) [16]. SSTT consists of side trunk movements in a seated position and does not require a great degree of hip joint motion. As trunk function is closely associated with gait function in community-dwelling older adults [17], SSTT has the potential to immediately improve gait speed for early postoperative THA patients.

As with gait speed, gait stability is an important assessment for gait function. Stride-time coefficient of variability (CV) is an indicator of gait stability and a useful predictor of fall risk [18]. The stride time CV of patients with THA one year after surgery does not improve compared to its preoperative level [19], and 36% of THA patients suffer a fall within 1 year after surgery [20]. Trunk function is critical for dynamic stability during gait [21] and the SST test primarily evaluates mobility function of the trunk. Therefore, SSTT also may improve the gait stability of THA patients.

This study aimed to examine the immediate effects of SSTT on gait function in early postoperative THA patients. The primary hypothesis was that SSTT would improve gait speed and stride time CV in patients undergoing THA. The secondary hypothesis was that SSTT does not change lower-extremity muscle strength despite improved gait function.

## 2. Materials and Methods

### 2.1. Participants

The participants were patients who underwent THA for unilateral hip osteoarthritis at a hospital between July and September 2018. In all participants, THA was performed with a direct anterior approach. Inclusion criteria were as follows: women with the ability to walk ≥20 m without assistance at measurement time, no medical or neurological diseases, and discharge on a clinical pathway (at 13 days). The study design was a quasi-randomized controlled trial. Participants were allocated into an intervention (SSTT) group or a control group in the order of the performed surgeries. Participants received an explanation of the aim of the study and provided written consent. The present study was conducted with the approval of the Institutional Review Board of university (approval no: 2018-102).

### 2.2. Measurement

To eliminate the effects of surgery as much as possible, immediate effects were assessed the day before discharge (post-operative day 12). The primary outcome measure was gait function, and the secondary outcome measure was lower-extremity muscle strength. Gait function and lower extremity muscle strength were measured before and after the interventions. The basic characteristics of the participants included age, height, body weight, body mass index, hip pain (visual analog scale), and Kellgren–Lawrence grade. The Kellgren–Lawrence grade was used to determine the degree of hip osteoarthritis [22]. Hip pain during gait was assessed with a visual analog scale; patients recorded their level of hip pain by drawing a vertical line between the ends of a 10 cm horizontal line [23]. Gait function was assessed by measuring comfortable gait speed, stride time, and stride time CV. Comfortable gait speed was measured on a 13 m walkway, and the initial and final 1.5 m sections were acceleration and deceleration zones. Measurement of stride time was based on a previous study; specifically, stride time was measured during a straight 20 m walk with angular rate sensors attached to affected limbs [24]. Stride time CV was calculated based on mean stride time and its standard deviation/mean × 100 (%) during the middle 15 m [25]. Comfortable gait speed and stride time were both measured twice, and the faster time of the two trials was recorded. Lower-extremity muscle strength was assessed by measuring hip abduction and knee extension using a handheld dynamometer. Hip abductor strength was measured in the supine position with both lower limbs in a neutral position [26]. The sensor pad of the handheld dynamometer was placed at the lateral femoral condyles. Knee extension strength was measured at a hip angle of 90° and with the knee flexed to 60° in the sitting position [26]. A strap was attached between the chair and a point on the patient’s ankle 5 cm above the lateral malleolus. The sensor pad was then placed at the front of the ankle under the strap to measure knee extension strength. Muscle strength measurement was performed twice, with a one-minute interval between trials, and the maximum value was used.

### 2.3. Interventions

**Figure 1:**
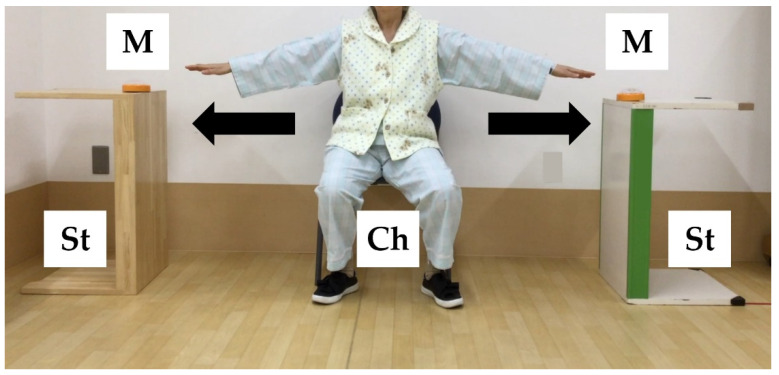
Movements of seated side tapping training. M: marker (diameter, 10 cm); Ch: chair (height, 41 cm from the floor); St: stand (height, 72 cm).

The intervention was based on a previous study [16]. Figure 1 shows the movements of SSTT. Briefly, participants abducted their shoulders 90° in a seated position and moved as quickly as possible to alternately touch markers located 10 cm from their fingertips to their left and right 10 times; participants performed 5 sets of this task. The SSTT group performed a single session of SSTT. The control group rested in a seated position for 10 min.

### 2.4. Statistical Analysis

Using G*Power 3.1, we calculated the sample size with an α of 0.05 and a Power (1 − β) of 0.80 with reference to the method described by Cohen et al. [27] and the effect size as stated in a previous study [16]. We assumed a dropout rate of 20%, and the necessary sample size for the present study was calculated as 20 participants per group. Student’s *t*-test, the chi-square test, or the Mann–Whitney U test was used to assess for significant differences in basic characteristics between the SSTT group and the control group. Pre–post-intervention comparisons were made by 2-way ANOVA. If an interaction was observed, the Bonferroni correction was used to correct for multiple comparisons. Effect sizes were calculated for items which demonstrated a significant difference. A *p*-value of <0.05 was considered to be statistically significant. All data were analyzed using IBM SPSS Statistics ver. 24.0 (IBM, Armonk, NY, USA).

## 3. Results

### 3.1. Basic Characteristics

**Figure 2:**
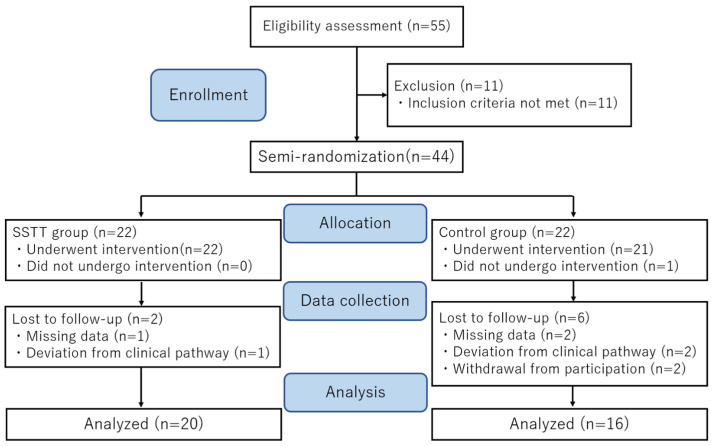
CONSORT flow diagram showing the enrollment and progress of the study participants.

Figure 2 shows the flow diagram of the process for enrollment in this study. The final analysis set consisted of 20 participants in the SSTT group and 16 participants in the control group. The basic characteristics of both groups are shown in Table 1. The mean ages of the participants in the SSTT and control groups were 66.5 ± 7.0 years and 62.6 ± 9.1 years, respectively. The two groups did not demonstrate any significant differences in basic characteristics.

### 3.2. Gait Function and Lower Extremity Muscle Strength

**Figure 3:**
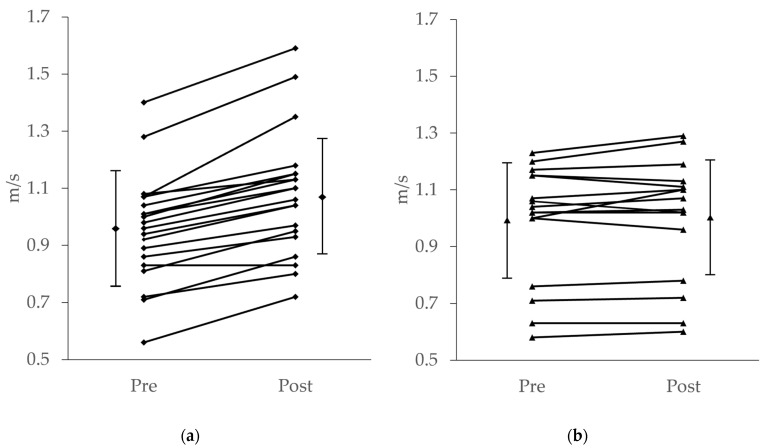
Gait speed of SSTT group (**a**) and control group (**b**) before and after the intervention. Group average and standard deviation (vertical bars). Individual data (solid lines).

In total, 95% (19/20) of participants in the SSTT group showed improved gait speed (Figure 3). Table 2 shows indicators of gait function and lower-extremity muscle strength. Interactions were observed among gait speed, stride time, and stride time CV; for all of these, the SSTT group showed significant pre–post-intervention improvement. In addition, gait speed, stride time, and stride time CV all demonstrated moderate to large effect sizes. No interaction was observed between hip abductor strength and knee extensor strength.

## 4. Discussion

In the present study, we aimed to examine whether a single-session SSTT, which focuses on high-velocity lateral trunk movement, immediately improves gait speed for in patients with THA. Although we observed no effect on lower-extremity muscle strength or pain, we did observe improvement in comfortable gait speed, stride time, and stride time CV. SSTT improved gait speed by 0.12 m/s. This improvement is considered a clinically relevant difference [28]. Thus, SSTT is an effective intervention for the improvement of gait speed for early postoperative THA patients.

The significant improvement in gait function of the THA patients may be mainly attributable to three factors. First, lateral trunk movement is a common determinant of gait and SSTT. Lateral trunk movement is necessary for maintaining balance during walking [17]. Similarly, quick lateral trunk movement in a seated position is related to gait speed [29]. Gait pattern in patients after THA is characterized by a lower lateral force rate and slow output of force, and this force output leads to a longer double support phase and slow gait speed [30]. Hodt-Billington et al. reported that trunk exercises in addition to the hip abductors may contribute to improving lateral stability during gait [31]. Trunk muscle exercises have been reported to improve gait speed in the elderly [32], patients with stroke [33], and patients after TKA [16]. The above suggests that SSTT, which consists of lateral movement of the trunk in a seated position, might help to improve lateral stability and gait function.

Second, movement velocity is important for gait speed. In general, increased muscle power is more closely associated with improved performance than increased muscle strength or muscle cross-sectional area [34]. Muscle power is expressed as the product of muscle strength and movement velocity. The fibers responsible for rapid contraction are selectively lost with age [35]. Muscle power is also reduced in patients undergoing THA for hip osteoarthritis, which is an age-related disease [36]. A study that focused on movement velocity found that unloaded high-velocity training yielded the same improvements in performance as conventional loaded training [37]. High-velocity training of hip muscles was also shown to improve gait speed and cadence in patients with hip osteoarthritis [38]. In the present study, SSTT was performed as quickly as possible post THA; consequently, lateral trunk movement velocity was significantly higher after the intervention. SSTT mainly focuses on the movement velocity of the trunk, and so this may have led to the improvement in gait speed.

Third, SSTT includes not only trunk movement but also hip movement. In our previous study of TKA patients, the SSTT group demonstrated a gait speed 0.11 m/s faster than that of the control group by three weeks of SSTT training [16]. We observed an immediate improvement in gait speed of 0.12 m/s in this study. Comparing the two studies, SSTT is likely to be more effective for patients undergoing THA than TKA. Based on these findings, because SSTT includes hip movements, it is considered that SSTT might improve hip movements and lead to enhancement of the speed.

On the other hand, CV—which is an indicator of gait regularity and pattern [39]—is believed to reflect adaptability to better forms of movement and reduced balance control [40]. It has been shown that CV is not improved following THA. For instance, in a previous study, CV was found to be high both preoperatively (3.3%) and at 3 months postoperative (2.8%) [18]. However, in the present study CV improved significantly from 2.3% before intervention to 1.8% after intervention. This result indicates that SSTT immediately improved not only gait speed, but also gait stability. Patients who underwent THA showed abnormal balance control, reflecting sensory and motor abnormalities [41]. SSTT, including head movement, may have contributed to sensory functions, including vestibular function [42] and improved gait stability.

The present study was limited in several ways. First, we did not evaluate trunk function during trunk training. Thus, it was unknown how the trunk function changed. Second, it assessed hip function in terms of abductor strength and did not assess other hip functions, such as range of motion and other aspects of hip muscle strength. Third, the study did not examine step length and cadence, which are determinants of gait speed, or changes in range of motion during gait, which are considered to affect stride time CV. Fourth, while SSTT demonstrated an immediate intervention effect, the absence of sufficient follow up means that the long-term effects of SSTT are unknown. It is necessary to verify the long-term effect and investigate the occurrence of possible adverse events, such as pain, if SSTT is used regularly.

## 5. Conclusions

In the present study, we examined the immediate effects of SSTT in early postoperative THA patients. Consequently, although there was no difference in hip pain and no improvement in muscle strength, significant improvements were observed in gait speed, stride time, and CV. These results suggest that SSTT may improve gait function immediately, easily, and safely in patients after THA.

## Figures and Tables

**Table 1:** Comparison of basic characteristics between SSTT and control groups.

	SSTT Group	Control Group	*p* Value
	n = 20	n = 16	
Age (y)	66.5 ± 7.0	62.6 ± 9.1	0.156
Height (m)	1.52 ± 0.05	1.55 ± 0.06	0.083
Body weight (kg)	55.0 ± 8.3	56.3 ± 9.5	0.650
Body mass index (kg/m^2^)	23.7 ± 3.7	23.1 ± 3.1	0.606
Kellgren–Lawrence grade III/IV	0/20	1/15	0.256
Visual Analog Scale	5.4 ± 7.9	6.3 ± 10.2	0.780

NOTE. Continuous data are expressed as the mean ± SD (Student *t*-test). Categorical data are expressed as n (chi-square test).

**Table 2:** Comparison of gait function and lower extremity muscle strength between SSTT and control groups.

		SSTT Group (n = 20)		Control Group (n = 16)		Interaction (Group × Time)	Effect Size (d)
		Pre	Post	Pre	Post		
Gait speed (m/s)		0.96 ± 0.2	1.08 ± 0.2 *	0.99 ± 0.2	1.00 ± 0.2	0.000	0.810
Stride time (s)		1.09 ± 0.2	1.02 ± 0.1 *	1.06 ± 0.1	1.05 ± 0.1	0.000	0.900
CV (%)		2.29 ± 0.9	1.76 ± 0.7 *	1.99 ± 0.5	2.00 ± 0.6	0.000	0.570
Hip abductor strength (Nm)							
	Operation side	21.4 ± 5.8	20.8 ± 6.0	19.5 ± 3.9	19.4 ± 3.7	0.879	
	Non-operation side	33.3 ± 7.8	33.7 ± 8.2	32.8 ± 7.4	32.7 ± 7.6	0.789	
Knee extensor strength (Nm)							
	Operation side	40.6 ± 12.0	40.2 ± 12.2	39.6 ± 9.3	38.8 ± 9.1	0.815	
	Non-operation side	65.6 ± 21.5	64.5 ± 20.7	64.3 ± 13.6	63.8 ± 13.5	0.765	

NOTE. Continuous data are expressed as the mean ± SD (post hoc test (Bonferroni) after two-way ANOVA). Abbreviations: SSTT, seated side tapping training; CV: stride time coefficient of variability. * Significant difference between pre- and post-SSTT.

## Data Availability

Data available on request due to restrictions, e.g., privacy or ethical concerns. The data presented in this study are available on request from the corresponding author.
